# LncRNA ENST00000539653 acts as an oncogenic factor via MAPK signalling in papillary thyroid cancer

**DOI:** 10.1186/s12885-019-5533-4

**Published:** 2019-04-02

**Authors:** Bin Song, Rurun Li, Zhihua Zuo, Juan Tan, Ling Liu, Dafa Ding, Yibing Lu, Dawei Hou

**Affiliations:** 1 Department of Endocrinology, The Second Affiliated Hospital of Nanjing Medical University, 121 Jiangjiayuan Road, Nanjing, 210011 China; 2 Department of Endocrinology, Clinical Medical College, Yangzhou University, 98 Nantong West Road, Yangzhou, 225001 China; 3 Department of Gerontology, Huai’an First People’s Hospital, Nanjing Medical University, 6 Beijing West Road, Huai’an, 223300 China; 4 Department of General Surgery, The Second Affiliated Hospital of Nanjing Medical University, 121 Jiangjiayuan Road, Nanjing, 210011 China

**Keywords:** Papillary thyroid cancer ∙ LncRNA ∙ proliferation ∙ biomarker ∙ MAPK

## Abstract

**Background:**

Papillary thyroid cancer (PTC) is the most frequent type of thyroid malignancy. In this study, we investigated the mechanisms whereby long non-coding RNAs (lncRNAs) are associated with PTC pathogenesis.

**Methods:**

Microarray analysis was used to determine differentially expressed lncRNAs between paired PTC tissues and normal adjacent thyroid tissues. Quantitative RT-PCR was used for validation in 86 PTC cases. Small interfering RNA (siRNA) transfection assays were then performed to assess how a novel lncRNA affected key proliferation and cell death pathways in IHH4 PTC cells.

**Results:**

We identified 1878 differentially expressed lncRNAs versus matched control samples (fold change ≥2.0, *P* < 0.05), of which 429 were upregulated and 1449 were downregulated. ENST00000539653.1 (ENS-653), one of the top hits in this microarray, was selected for further study. Higher ENS-653 expression was observed in PTC tissue samples versus adjacent normal tissues, and was associated with a larger tumor size and a more advanced clinical stage. In the Cancer Genome Atlas (TCGA) PTC cohort, higher ENS-653 expression was correlated with more frequent BRAF (V600E) mutation and poorer disease-free survival. Furthermore, ENS-653 downregulation reduced the proliferation of PTC cells and led to G1-S arrest, but had no impact on apoptosis. ENS-653 downregulation also inactivated ERK1/2 and ERK5, causing partial MAPK cascade suppression.

**Conclusion:**

ENS-653 exhibits oncogenic properties in PTC, and could be a diagnostic and/or prognostic PTC biomarker, in addition to possibly being a future target for therapy.

**Electronic supplementary material:**

The online version of this article (10.1186/s12885-019-5533-4) contains supplementary material, which is available to authorized users.

## Background

Thyroid cancer is the most common form of endocrine tumors, and its incidence has increased rapidly worldwide recently [1, 2]. Papillary thyroid cancer (PTC), which makes up roughly 80% of thyroid cancers, is the principal histologic type of this cancer [3]. Most PTC patients are curable and have a favorable prognosis with the current therapeutic regimen that includes surgical resection, thyroid hormone suppression and radioactive iodine therapy [4]. Nevertheless, a small proportion of PTC cases have a poor prognosis due to metastases, and it is often challenging to differentiate these patients from the lower risk cases at the early stage. Thus, identification of informative biomarkers could aid in molecular stratification of aggressive and indolent PTC.

Over the past decade, several molecular markers, including *RAS*, *RET-PTC*, and *BRAF* (V600E) gene mutations, have been linked with PTC [5]. Other driver mutations include *EIF1AX*, *PPM1D* and *CHEK2*, as documented in the Cancer Genome Atlas (TCGA) which contains datasets for 496 PTC cases [6]. In addition, microRNAs have been found to be important contributors to the oncogenesis of PTC [7].

Long non-coding RNAs (lncRNAs), which are at least 200 nucleotides long, do not code for any protein. Accumulating evidence has increasingly implicated lncRNAs in cancer development and/or progression across a range of tumor types including PTC [8]. This has led to a surge of interest in lncRNAs as prognostic or diagnostic cancer biomarkers [9–13]. In several instances, specific lncRNAs have been demonstrated to participate in PTC oncogenesis and progression. Xu et al. have shown that lncRNAs ENST00000537266 and ENST00000426615 are upregulated in PTC tissue and impact PTC cell proliferation and motility [14]. PTC susceptibility candidate 3 (PTCSC3) governs PTC cell proliferative and migratory capacity via S100A4 [15] and the Wnt/β-catenin pathway [16]. BRAF-activated non-coding RNA (BANCR) may also be a prognostic marker of PTC [17]. Furthermore, lncRNAs GAS8-AS1 and LPAR4 were identified by whole exome sequencing as novel driver alterations in PTC [18]. However, the precise molecular mechanisms underlying how lncRNAs affect PTC remain poorly explored.

In the current study, we sought to identify differentially expressed lncRNAs in paired PTC tissues and adjacent normal tissue samples by using an lncRNA microarray and examined the functional role of lncRNA ENST00000539653.1 (ENS-653) in PTC oncogenesis.

## Methods

### Tissue acquisition

Fresh surgical specimens were collected from 86 patients with pathologically proven PTC, snap frozen in liquid nitrogen and stored at − 80 °C. These patients underwent surgery at the Department of General Surgery, the Second Affiliated Hospital of Nanjing Medical University, Nanjing, China, between January, 2016 and June, 2018. Major inclusion criteria were: (1) patients with pathologically confirmed PTC in the primary tumor and without any severe diseases in other organs; (2) patients that had received total/near total thyroidectomy and had not received any radiotherapy; (3) patients with a negative history of any other malignant tumors. Major exclusion criteria were: (1) patients with a positive history of other malignant tumors; (2) patients diagnosed with histological types of thyroid cancer other than PTC; (3) patients with severe diseases such as heart failure, stroke, and chronic renal failure; (4) patients with a history of ^131^I therapy. The study flowchart is shown in Additional file 2: Figure S1 and patient demographic and baseline characteristics are shown in Additional file 1: Table S1. The tumor clinical stages were based on the American Joint Committee on Cancer (AJCC) TNM system [19].

The study protocol was approved by the local ethics committee at the authors’ affiliated hospital. Written informed consent was provided by all participants before any sample or data collection.

### RNA extraction

Total cellular RNA was isolated using Trizol reagent (Invitrogen, Carlsbad, CA, USA) and purified using RNase-Free DNase (Invitrogen) as described by the manufacturer. RNA concentration and purity were determined using a NanoDrop 2000 (Thermo Scientific, Wilmington, DE, USA).

### LncRNA microarray expression profiling

Total RNA from four PTC samples and matched adjacent normal thyroid tissues was isolated by TRIzol reagent (Invitrogen) and further purified using a commercially available kit (QIAGEN, GmbH, Germany). The demographic and baseline characteristics of these four patients are shown in Additional file 1: Table S2. RNA was then quantified using a spectrophotometer, and its integrity was evaluated by Agilent 2100 (Agilent Technologies, Santa Clara, CA, USA). Each purified RNA sample was reverse transcribed into double strand cDNA, and then synthesized into cRNA. The cRNAs labeled with Cy3-dCTP were processed and hybridized onto a 4 × 180 K human LncRNA Microarray V4.0 (Agilent Technologies), which is designed for global profiling of 40,916 lncRNAs. The microarray was conducted by Capitalbio Technology Corp, Beijing, China, based on the manufacturer’s instructions. Each transcript was accurately identified by a specific exon or splice junction probe. The hybridized arrays were subsequently washed, fixed and scanned using a Microarray Scanner (Agilent Technologies), and the Feature Extraction software (Agilent Technologies) was used for raw data collection. Data analysis was performed with GeneSpring software V13.0 (Agilent Technologies). Differential expression was identified on the basis of both a ≥ 2 and < − 2-fold change threshold and a corrected *P* value < 0.05.

### Cell culture and siRNA transfection

Human PTC cell line IHH4 was a gift from Professor Chongyou Lu (Chinese University of Hong Kong, Hong Kong, China). Cells were cultured at 37 °C in an incubator containing 5% CO_2_ in RPMI 1640 with 10% fetal bovine serum (FBS), 1% non-essential amino acids, and 100 U/mL penicillin and streptomycin. IHH4 cells were grown to 30–50% confluence and transfected with 100 nmol/L Si-653 (siRNA silencing ENS-653, GenePharma, Shanghai, China) or Si-NC (negative control) using Lipofectamine 2000 (Invitrogen) based on the manufacturer’s instructions. The sequences of Si-653 are shown in Additional file 1: Table S3. Non-transfected control cells were treated only with Lipofectamine 2000 (Blank).

### Cell counting Kit-8 (CCK-8) assays

CCK-8 assays (Dojindo, Japan) were performed as instructed by the manufacturer. Briefly, 8 × 10^3^ cells were plated in a 96-well plate. After incubation for 1 to 4 d, 10 μL CCK-8 solution was added to each well. After incubation for 4 h at 37 °C, the results were read at 450 nm using a microplate reader. The experiments were performed at least three times independently in triplicate.

### Colony formation assays

Transfected cells were plated in 10 cm dishes and were fixed in 1% paraformaldehyde after incubation for 6 d. After staining with 0.1% crystal violet, colonies (> 50 cells) were enumerated and imaged under a light microscope. The experiments were performed at least three times independently in triplicate.

### Flow cytometry

Transfected cells were stained with annexin V-PE and 7-AAD using a commercially available apoptosis detection kit as instructed by the manufacturer (BD Biosciences, Mississauga, ON, Canada). For cell cycle analysis, a flow cytometer (BD Biosciences) was used to assess the percentage of apoptotic cells or cell cycle distributions via the CellQuest software (BD Biosciences). The experiments were performed at least three times independently in triplicate.

### Quantitative reverse transcription (qRT-) PCR

HiScript Q RT SuperMix (Vazyme Biotech) was used for cDNA reverse transcription from isolated RNA as described by the manufacturer. The sequences of primers for lncRNAs (Invitrogen) are shown in Additional file 1: Table S3. The SYBR Green RT-PCT Kit (Vazyme Biotech) was used for all qRT-PCR assays, which were run in a 10 μL total volume with 0.2 μL 10 μmol/L forward/reverse primers, 3.6 μL RNase-free ddH_2_O, 1 μL cDNA, and 5 μL SYBR Green Master Mix. The PCR was run at 95 °C for 10 min followed by 40 cycles of 95 °C for 10 s, and 60 °C for 1 min. *GAPDH* was the normalization control. Relative lncRNA expression was established using the delta-delta Ct (ΔΔCt) method.

### Western blotting assays

IHH4 cells were lysed using a lysis buffer containing fresh protease inhibitors (Beyotime Biotechnology, Shanghai, China). The protein concentrations of the lysate were determined using the BCA method (Beyotime). Primary antibodies (at 1:1000 dilution unless otherwise indicated) against the following proteins were used for immunoblotting assays: β-actin (#2872; CST, Beverly, MA, USA), PCNA (ab92552; Abcam, Cambridge, UK), cyclin D1 (ab134175; Abcam), cleaved caspase-3 (#29034; SAB, College Park, MD, USA), ERK1/2 (ab184699; Abcam), phospho-ERK1/2 (ab214362; Abcam), ERK5 (ab40809; Abcam), phospho-ERK5 (ab5686; Abcam), JNK (ab208035; Abcam), phospho-JNK (ab124956; Abcam), p38 (#8690; CST) and phospho-p38 (#4511; CST). HRP-conjugated goat anti-rabbit secondary IgG (1:3000) (KeyGen Biotech, Nanjing, China) were used to probe blots at room temperature for 1 h. A Tanon-4500 system (Tanon Science and Technology, Shanghai, China) was used for digital blot visualization.

### RNA-Seq data analysis in TCGA PTC cohort

We downloaded RNA-Seq expression values for ENS-653 (ENSG00000250748) of 497 TCGA PTC patients and 59 matched normal samples from TANRIC data base (https://ibl.mdanderson.org/tanric/_design/basic/index.html). For ENS-653 expression analysis, only PTC with a matched normal sample was used. Additionally, we downloaded corresponding clinicopathologic data from TCGA dataset from the website of The cBioPortal for Cancer Genomics (http://www.cbioportal.org/). Combined with RNA-Seq expression values from TANRIC data base, we analyzed the relationship between ENS-653 expression and PTC clinicopathologic characteristics. For correlation analysis, only the tumor samples were used.

### Statistical analysis

Continuous data were reported as mean ± standard deviation (SD) or median (interquartile range, IQR), while categorical data were reported as count and percentage. Error bars in the scatter plots and the bar graphs represent SD or IQR. Chi-squared tests or Fisher exact tests were used as appropriate for comparison of categorical data between groups. Continuous data were examined whether they were normally distributed with the One-Sample Kolmogorov-Smirnor test. If the data were normally distributed and the variation between groups was comparable, the comparisons of measurement data between two groups were performed using the paired-sample t test or independent-sample t test. The comparisons among three or more groups were firstly performed by One-Way ANOVA test if the variance between groups were comparable. When the data showed skewed distribution, comparisons were performed by nonparametric Mann-Whitney tests. Regression analysis (unadjusted or adjusted) was used to evaluate independent factors associating with ENS-653 level in TCGA PTC cohort. Disease-free survival was evaluated by the Kaplan–Meier survival curve and the Log-rank test. The GraphPAD Prism 5 software (GraphPad Software Inc., USA) or EmpowerStats (http://www.empowerstats.com/cn/; X&Y Solutions, Boston, MA) was used for all analyses. Statistical tests were two-sided and *P* < 0.05 was considered statistically significantly different.

## Results

### LncRNA expression in PTC tissues

**Fig. 1:**
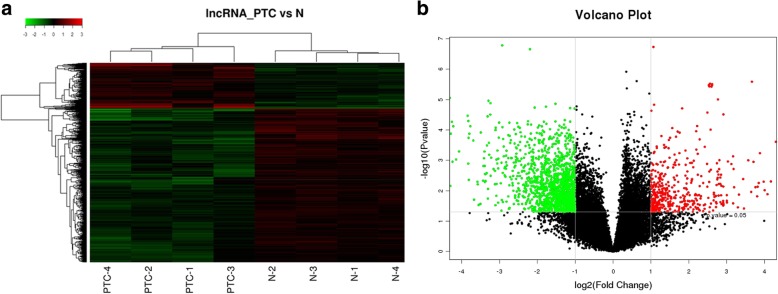
LncRNA microarray analysis in papillary thyroid cancer (PTC) tissues compared with matched adjacent noncancerous thyroid tissues. **a** Hierarchical clustering analysis of differentially expressed lncRNAs. Red and green colors indicate high and low expression, respectively. In the heat map, columns represent samples and rows represent each lncRNA. **b** Volcano plot of differentially expressed lncRNAs between PTC and paired noncancerous thyroid tissue. The vertical lines correspond to 2.0-fold upregulation and downregulation, and the horizontal line represents a *P* value of 0.05

Relative to paired normal adjacent tissues, lncRNA microarray analysis identified a total of 1878 (4.67%) differentially expressed lncRNAs in PTC tissues; 429 (1.05%) lncRNAs were upregulated and 1449 (3.54%) lncRNAs were downregulated. These hits were grouped based on expression levels via hierarchical clustering (Fig. 1a). A volcano plot presenting the identified lncRNAs is shown in Fig. 1b.

### Microarray data validation

**Fig. 2:**
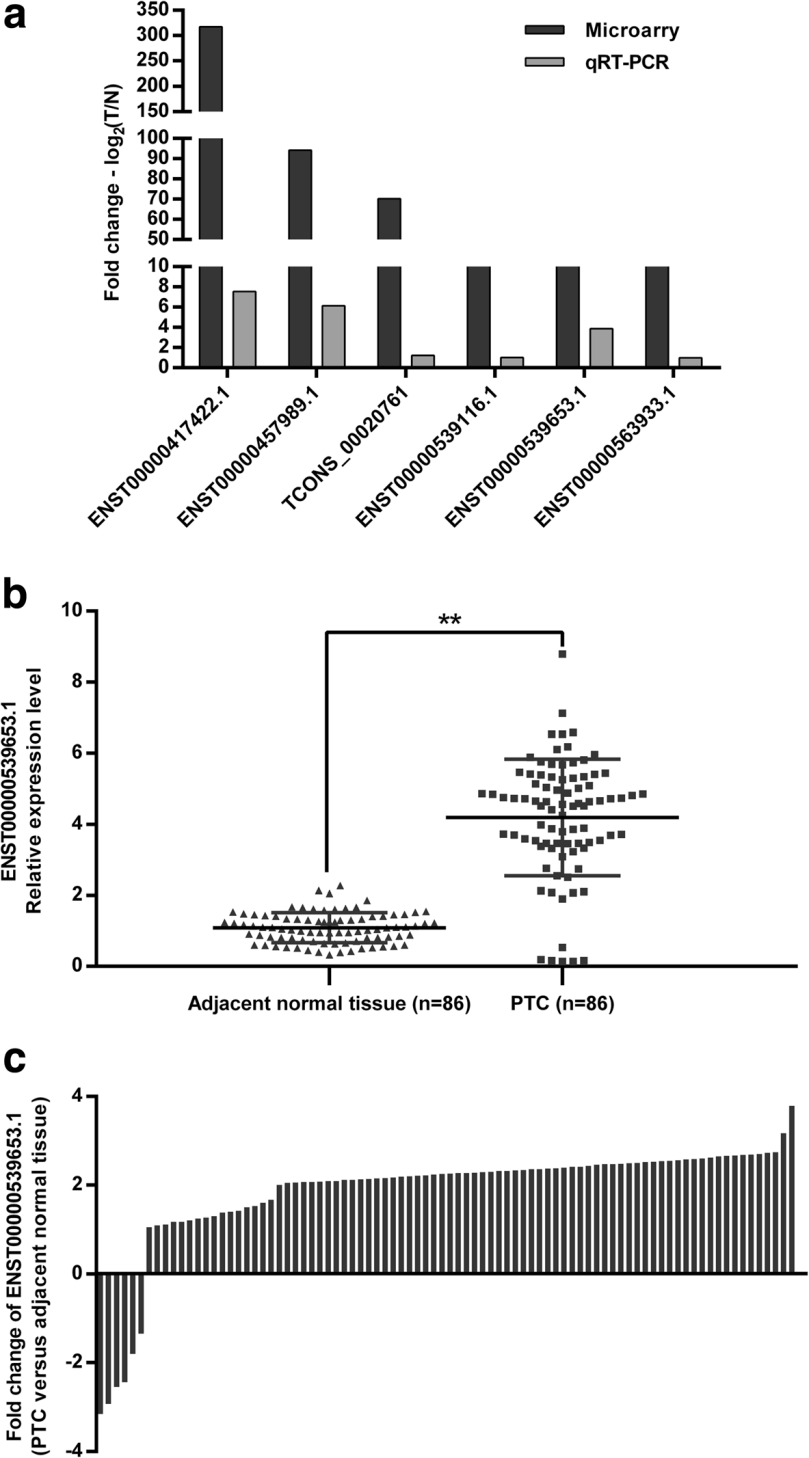
QRT-PCR validation of selected differentially expressed lncRNAs. **a** Comparison between microarray and qRT-PCR results. The heights of the columns represent the log-transformed median fold changes (Tumor/Normal tissues) in the expression in 86 paired PTC and noncancerous thyroid tissues. **b** Relative expression level of ENS-653 in 86 pairs of PTC and noncancerous thyroid tissues. ENS-653 expression was evaluated by qRT-PCR and normalized to GAPDH mRNA expression. Data are expressed as a mean ± SD. **c** Proportion of cases with positive fold changes of ENS-653 expression in PTC compared with noncancerous thyroid tissues. **P < 0.01

The top 10 differentially expressed lncRNAs from the microarray were selected for further analysis (Table 1). To select relevant lncRNAs for validation, we consulted the UCSC database (http://genome.ucsc.edu) to filter the lncRNAs based on documented abundance in thyroid tissues as assessed via RNA-seq. Six overlapping lncRNAs (ENST00000417422.1, ENST00000457989.1, TCONS_00020761, ENST00000539116.1, ENST00000539653.1 and ENST00000563933.1) were selected and qRT-PCR was used to further confirm their expression in 86 paired PTC and adjacent normal thyroid tissues. These results revealed that, consistent with their upregulation in lncRNA microarrays, all the 6 lncRNAs showed higher expression levels in PTC tissues versus normal adjacent tissues and the levels of ENST00000417422.1, ENST00000457989.1 and ENST00000539653.1 were significantly higher in PTC tissues versus normal adjacent tissues (*P* < 0.05) (Fig. 2a). ENST00000417422.1 and ENST00000457989.1 were reported to be involved in PTC [19, 20]. However, the role of lncRNA ENST00000539653.1 (ENS-653), which was significantly upregulated in PTC versus adjacent normal tissues (*P* < 0.01) (Fig. 2b, c), is largely unknown. Therefore, we selected ENS-653 located on chromosome 12 (Additional file 2: Figure S2) for further investigation.

**Table 1:** Top 10 aberrantly expressed lncRNAs in microarray

LncRNA ID	Database	Fold change	Regulation	P value
ENST00000417422.1	ENSEMBL	317.4575	up	0.000031
ENST00000457989.1	ENSEMBL	94.12319	up	0.000003
XR_429125.1	RefSeq	89.38535	up	0.000109
TCONS_00020761	HumanLincRNACatalog	70.23221	up	0.000251
HIT000218960	H-InvDB	64.69215	up	0.006213
ENST00000547051.1	ENSEMBL	48.38944	down	0.000155
uc021ssa.1	UCSC	43.16758	up	0.000025
ENST00000539116.1	ENSEMBL	40.02352	up	0.002246
ENST00000539653.1	ENSEMBL	39.99428	up	0.001013
ENST00000563933.1	ENSEMBL	35.21136	up	0.000805

### High ENS-653 expression is associated with larger tumor size and more advanced tumor stage

**Fig. 3:**
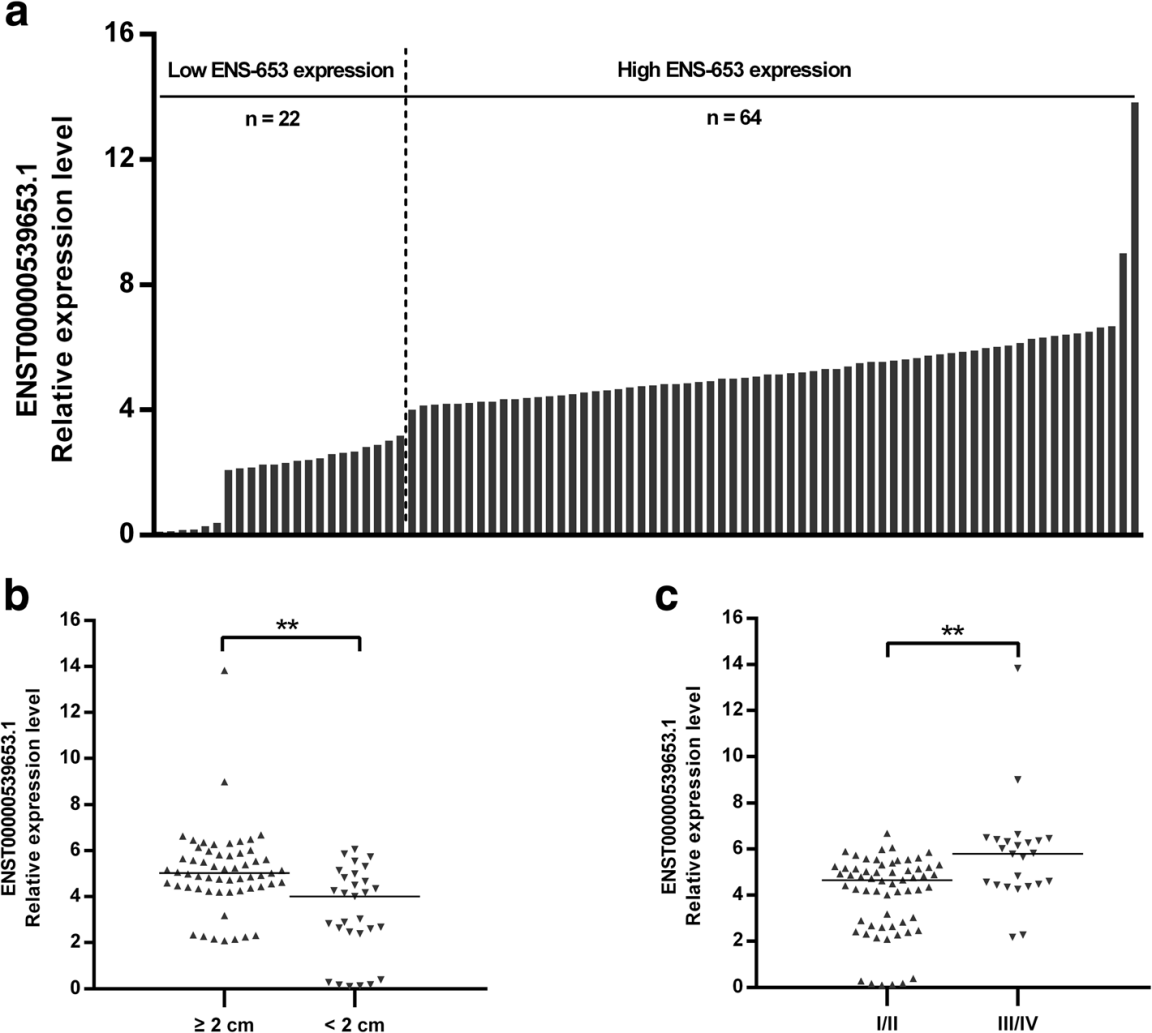
Association of ENS-653 expression with tumor size and stages in PTC patients. **a** ENS-653 expression was classified into two groups. Final results are presented as fold change in tumor tissues relative to normal tissues. Fold change is greater than or equal to 2.0 for high expression, and less than 2.0 for low expression. **b**, **c** ENS-653 upregulation was associated with larger tumor size and advanced stages. Data are expressed as median, **P < 0.01

The mean expression levels of ENS-653 were 4.5 ± 2.0 (range: 0.1 to 13.8) (Fig. 3a). To evaluate whether ENS-653 expression was associated with clinicopathologic features of PTC patients, we categorized 86 PTC patients into the high ENS-653 expression group (*n* = 64; fold-change ≥2) and the low ENS-653 expression group (*n* = 22; fold-change < 2). Significantly more patients in the high expression group had a tumor size ≥2 cm (76.6% vs. low expression: 36.4%; *P* < 0.001) and a more advanced Clinical stage (40.6% vs. low expression: 9.1%; *P* < 0.001) (Table 2); Meanwhile, patients with tumor size ≥2 cm, as well as with advanced Clinical stages had a higher expression of ENS-653 (*P* < 0.01) (Fig. 3b and c).

**Table 2:** Correlation of ENS-653 expression of with clinicopathologic variables of PTC patients

Characteristics	ENS-653 expression^a^		*P-value*
	Low	High	
	(*n* = 22)	(*n* = 64)	
Age (years)	51.5 ± 13.2	57.2 ± 13.3	0.088^*^
Gender [n(%)]			0.833^†^
Female	16 (72.7)	48 (75.0)	
Male	6 (27.3)	16 (25.0)	
Histological subtypes [n(%)]			1.000^§^
Classic	21 (95.5)	62 (96.9)	
Follicular	1 (4.5)	2 (3.1)	
Tumor size [n(%)]			< 0.001^†^
< 2 cm	14 (63.6)	15 (23.4)	
≥2 cm	8 (36.4)	49 (76.6)	
Extrathyroidal extension [n(%)]	2 (9.1)	7 (10.9)	1.000^§^
Multifocality [n(%)]	6 (27.3)	17 (26.6)	0.948^†^
Coexistent HT^b^ [n(%)]	5 (22.7)	15 (23.4)	0.946^†^
T Stage [n(%)]			0.856^§^
T1	14 (22.7)	15 (17.2)	
T2	7 (72.7)	46 (78.1)	
T3	1 (4.5)	2 (3.1)	
T4	0 (0.0)	1 (1.6)	
N1 [n(%)]	9 (40.9)	27 (42.2)	0.916^†^
M1 [n(%)]	0 (0.0)	4 (6.2)	0.568^§^
Clinical Stage [n(%)]			0.001^§^
I	11 (50.0)	8 (12.5)	
II	2(9.1)	26(40.6)	
III	8(36.4)	25(39.1)	
IV	1 (4.5)	5 (7.8)	

^a^Fold change(FC) (tumor tissues relative to normal tissues) is greater than or equal to 2.0 for high expression, and less than 2.0 for low expression

^b^Hashimoto’s thyroiditis

^*^P values were calculated by Student’s *t* test

^†^*P* values were calculated by χ^2^ test

^§^*P* values were calculated by Fisher exact test

### High ENS-653 expression is associated with BRAF(V600E) mutation and poorer disease-free survival in TCGA PTC cohort

**Fig. 4:**
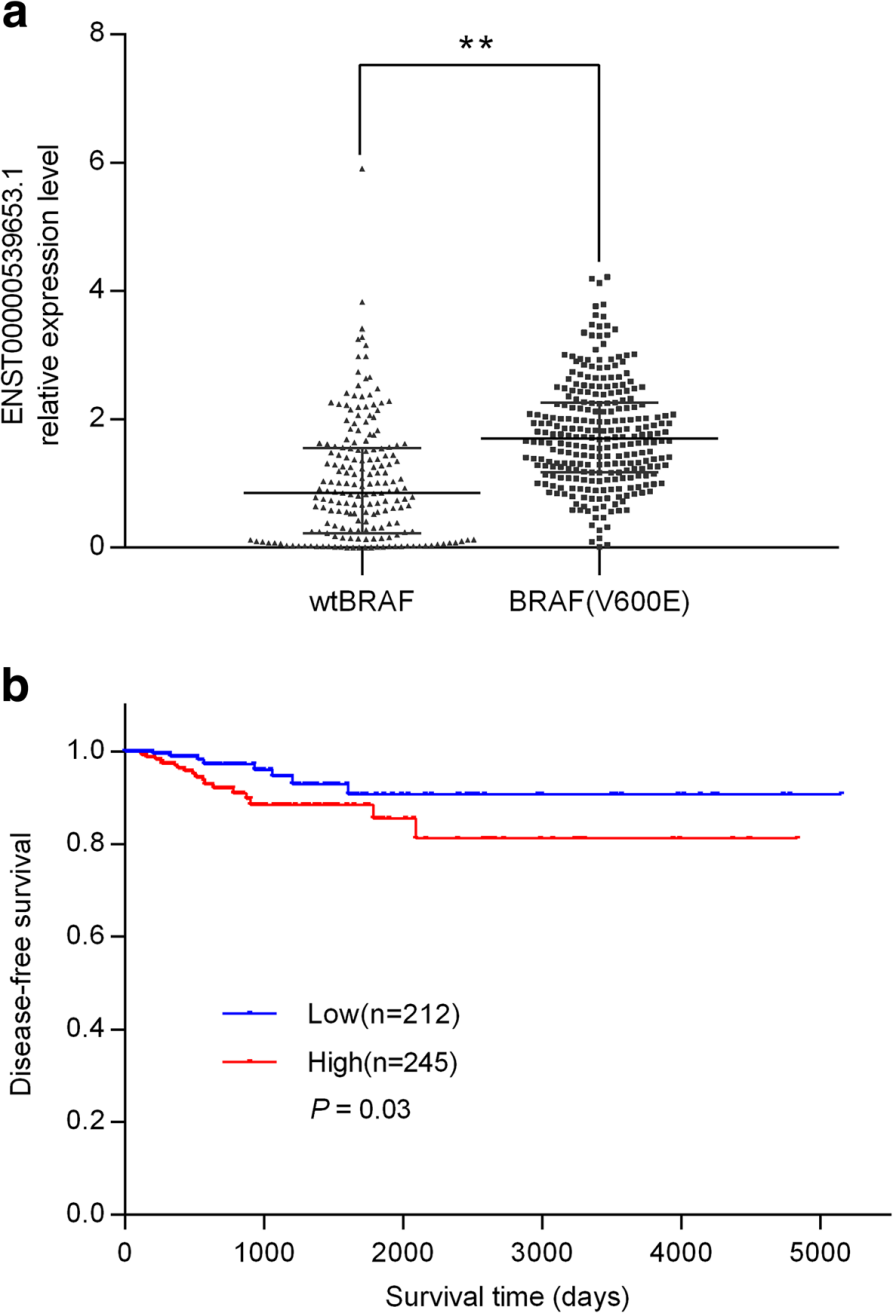
Association of ENS-653 expression with BRAF (V600E) mutation and disease-free survival in TCGA PTC cohort. **a** Statistical analysis of ENS-653 expression between BRAF (V600E) mutation and wtBRAF PTC patients. Data are expressed with median (interquartile range). **P < 0.01. **b** Survival was analyzed and compared between patients with high and low levels of ENS-653 expression; *n* = 457, log-rank test

The median expression level of ENS-653 was significantly higher in PTC samples than that in matched normal tissues (0.289 vs. 0.004; *P* < 0.001). Using median, we divided 462 PTC patients into the high ENS-653 expression group (*n* = 231) and the low ENS-653 expression group (*n* = 231). Significantly more patients in the high expression group had BRAF (V600E) mutation (73.7% vs. low expression: 42.4%; *P* < 0.001) (Additional file 1: Table S4); Meanwhile, patients with BRAF(V600E) mutation had a higher expression of ENS-653 (*P* < 0.01) (Fig. 4a). Moreover, regression analysis suggested that BRAF (V600E) mutation was also associated with higher ENS-653 after adjustment for gender and age (Additional file 1: Table S5). In addition, high levels of ENS-653 expression were significantly associated with poorer disease-free survival (Fig. 4b).

### ENS-653 downregulation suppresses PTC cell proliferation

**Fig. 5:**
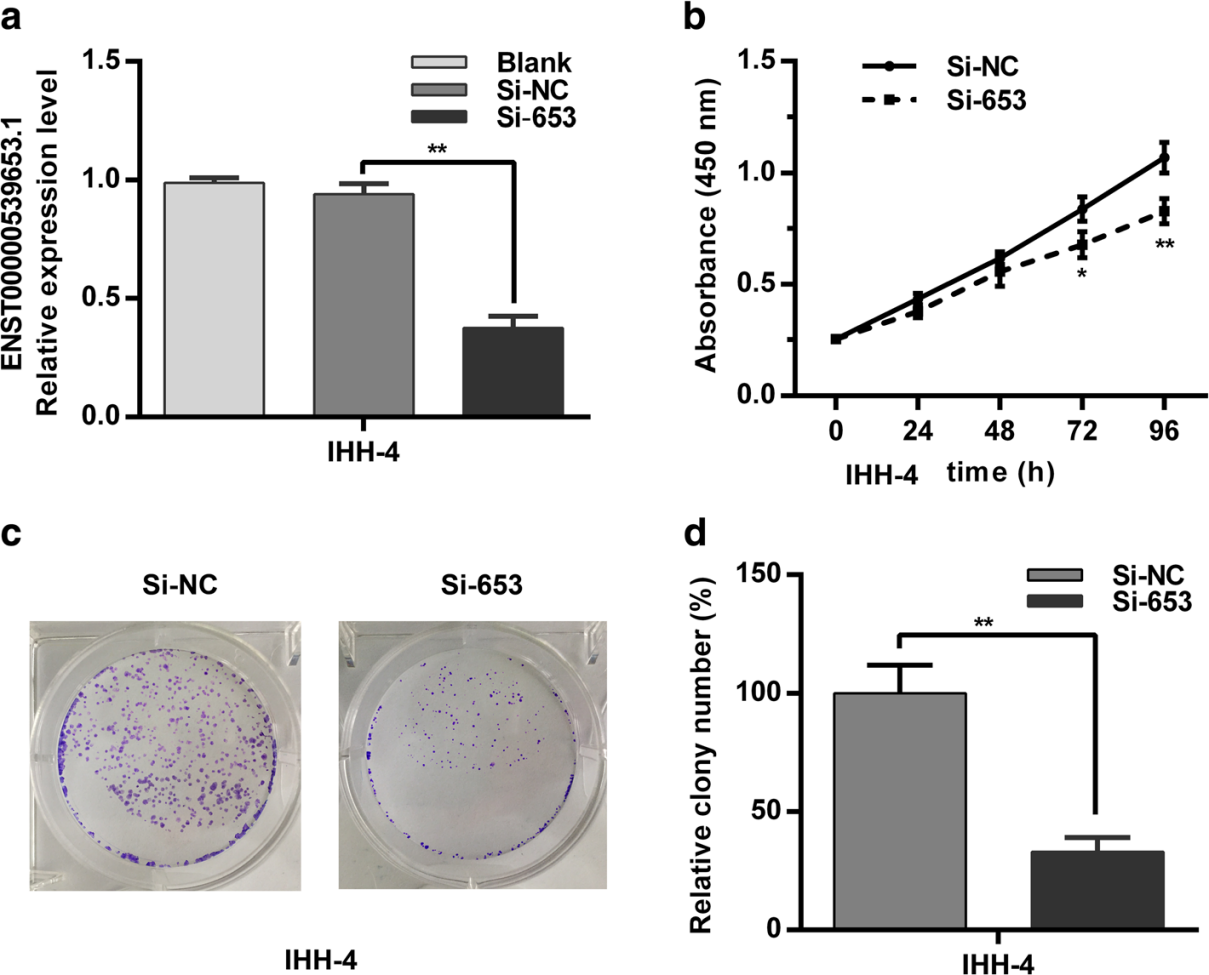
ENS-653 downregulation suppresses PTC cell proliferation. **a** qRT-PCR of IHH4 cells transfected with siRNA silencing ENS-653 (Si-653) or negative control (Si-NC). **b** CCK-8 assays were performed in Si-653 or Si-NC transfected IHH4 cells. Values represent mean ± SD from three independent experiments in triplicate. **c** Colony forming assays were performed in Si-653 or Si-NC transfected IHH4 cells. **d** Relative colony numbers of IHH4 cells transfected with Si-653 or Si-NC. Values represent mean ± SD from three independent experiments in triplicate. *P < 0.05, **P < 0.01

We next examined the role of ENS-653 expression in cancer cell proliferation. We first knocked down ENS-653 expression in IHH4 cells by transfection with Si-653 (Fig. 5a). CCK-8 assays revealed that ENS-653 downregulation suppressed proliferation of IHH4 cells at 72 h and 96 h post transfection compared with controls (Fig. 5b). Colony formation assays demonstrated that ENS-653 downregulation significantly reduced the number of colonies by IHH4 cells versus cells transfected with Si-NC (*P* < 0.01) (Fig. 5c and d). These findings indicate that ENS-653 may regulate the proliferation of PTC cells.

### ENS-653 downregulation induces a G1 cell cycle arrest

**Fig. 6:**
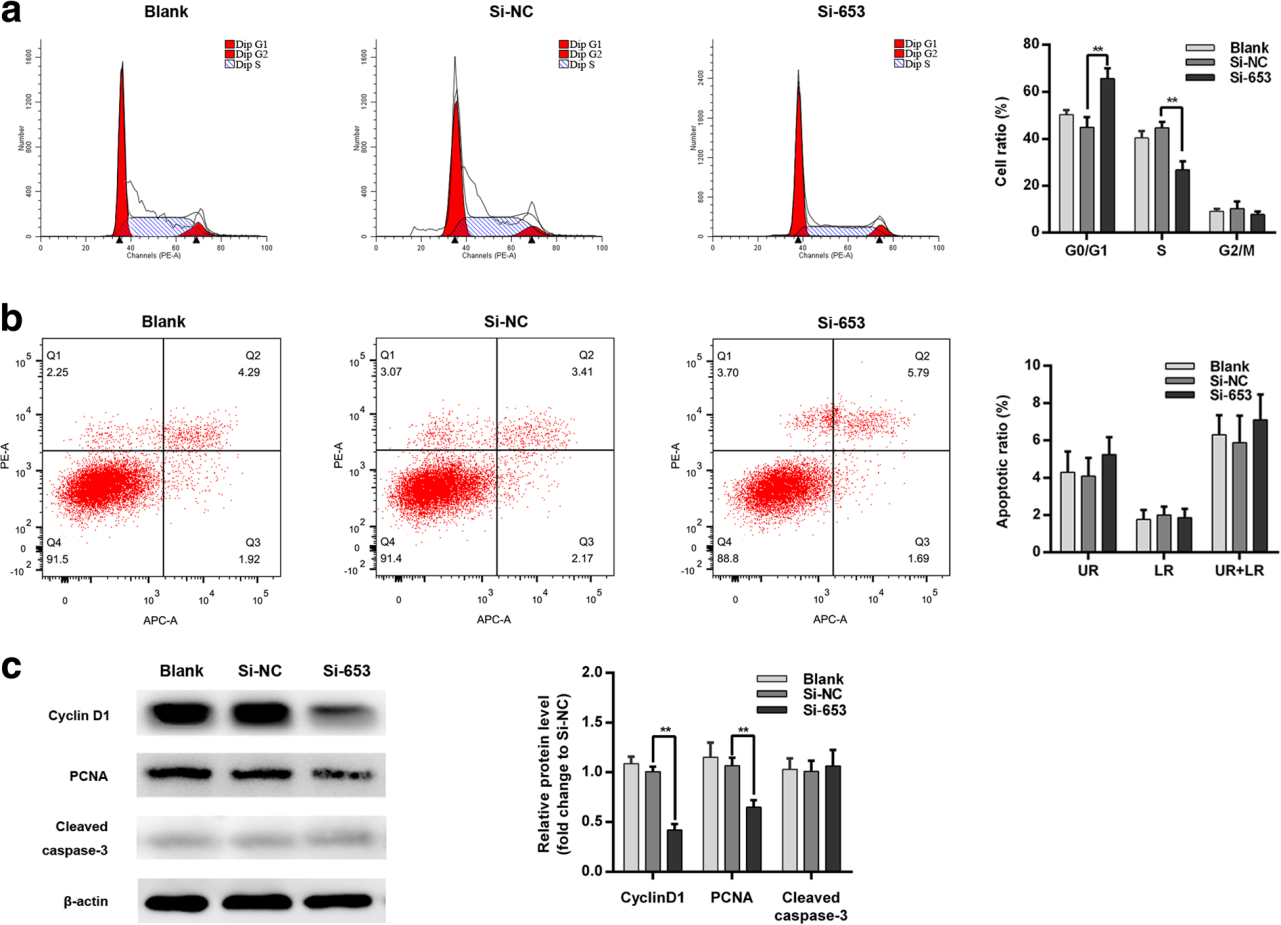
ENS-653 downregulation induces a G1 cell cycle arrest with no impact on apoptosis. **a** Flow cytometry was performed in IHH4 cells transfected with Si-653 (siRNA silencing ENS-653) or Si-NC (negative control). The bar chart represents the percentage of IHH4 cells in G0/G1, S or G2/M phase. **b** Flow cytometry was used to detect the apoptotic cells in IHH4 cells transfected with Si-653 or Si-NC. LR, early apoptotic cells; UR, terminal apoptotic cells. Values represent mean ± SD from three independent experiments. **P < 0.01. **c** Western blot analysis of PCNA, cyclin D1 and cleaved caspase-3 in Si-653 or Si-NC transfected and IHH4 cells. β-actin was used as a loading control

We next investigated how ENS-653 downregulation affected cell cycle distributions and apoptosis of IHH4 cells. Flow cytometry revealed a significantly increased proportion of IHH4 cells in the G0/G1 phase with a remarkable decrease in the S phase in Si-653-transfected IHH4 cells versus Si-NC (*P* < 0.01) (Fig. 6a). Consistently, our immunoblotting assays showed that ENS-653 downregulation was associated with lower levels of PCNA and cyclin D1 (Fig. 6c). Furthermore, we found no apparent differences in the apoptotic rate of IHH4 cells transfected with Si-653 and Si-NC (*P* > 0.05) (Fig. 6b). Western blotting assays showed no significant difference in the levels of cleaved caspase-3 between IHH4 cells transfected with Si-653 and those transfected with Si-NC (*P* = 0.65) (Fig. 6c). These results show that ENS-653 induces changes in cell cycle distributions of IHH4 cells, but has no effect on apoptosis of IHH4 cells.

### Interference with ENS-653 partially suppresses MAPK signaling

**Fig. 7:**
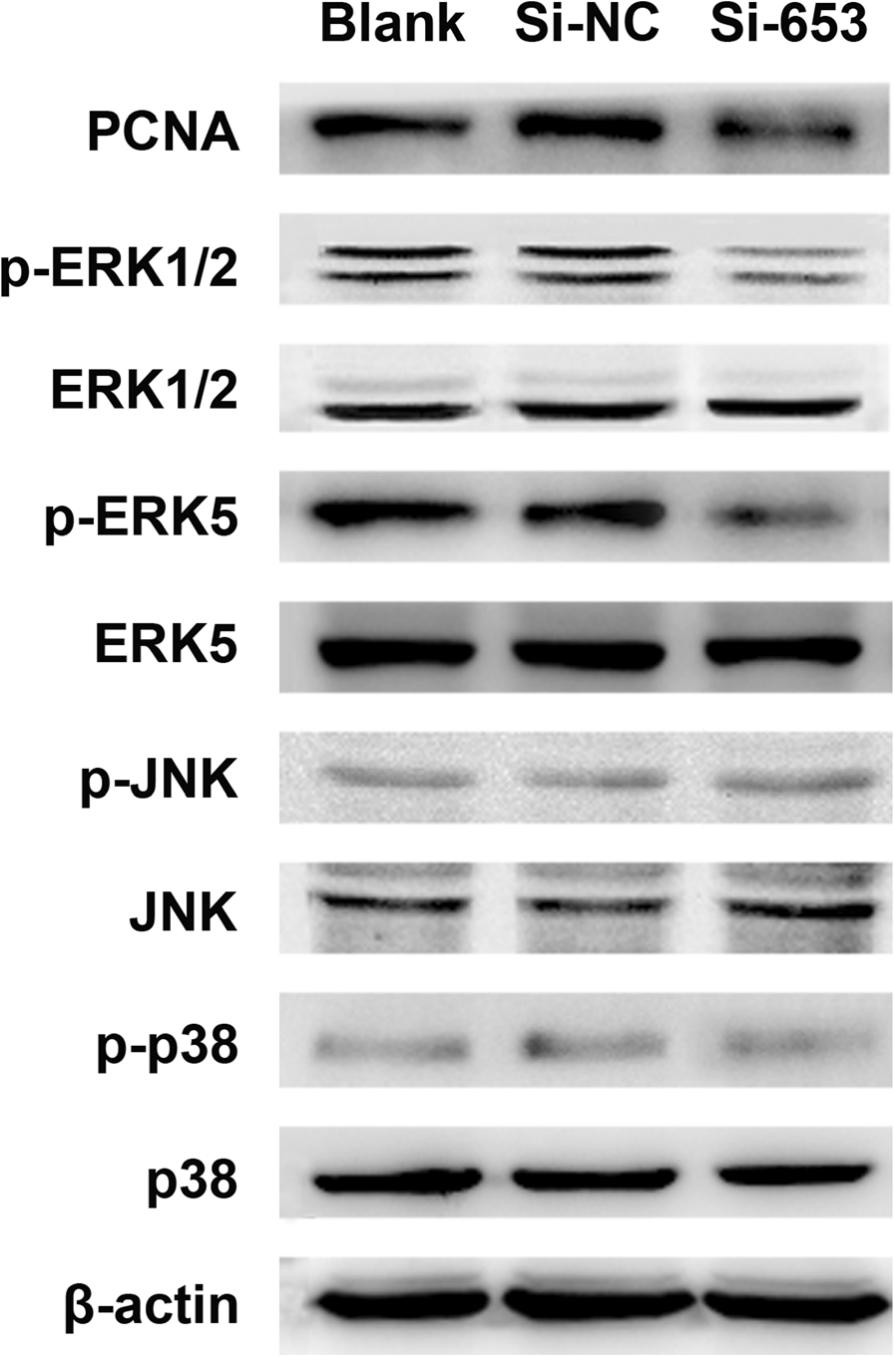
Interference with ENS-653 causes partial MAPK cascade suppression. Western blot analysis of total and phosphorylated ERK1/2, ERK5, JNK and p38 in IHH4 cells transfected with Si-653 (siRNA silencing ENS-653) or Si-NC (negative control)

To assess the link between MAPK signaling and ENS-653-induced IHH4 cell proliferation, we investigated the expression and phosphorylation status of ERK1/2, ERK5, JNK and p38 in Si-653-transfected IHH4 cells and Si-NC-transfected cells (Fig. 7). ERK1/2 and ERK5 phosphorylation was downregulated in Si-653-transfected cells compared with Si-NC-transfected cells, while the total ERK1/2 and ERK5 levels did not change. In contrast, total and phosphorylated JNK or p38 levels did not change upon ENS-653 downregulation. Together these findings indicate that interference with ENS-653 causes partial MAPK cascade suppression.

## Discussion

Much evidence indicates that lncRNAs play key roles in diverse human cancers [8–10, 12], including PTC [14–18, 21]. LncRNAs can function as tumor-promoting or tumor-suppressing factors by affecting key processes such as migration, invasion, proliferation, and apoptosis during thyroid cancer pathogenesis [22]. LncRNAs may therefore have great potential as prognostic and/or diagnostic biomarkers of thyroid cancer progression, in addition to being viable candidate therapeutic targets [22].

In this study, we discovered via microarray that many lncRNAs were aberrantly expressed in PTC tissues, indicating the important role of lncRNAs in PTC. We selected a novel lncRNA (ENS-653), which was previously uncharacterized, for further analysis. Our results demonstrated that expression of ENS-653 was markedly increased in PTC tissues, and higher ENS-653 expression was associated with a larger tumor size and a more advanced clinical stage. In addition, using TCGA PTC cohort, we found higher ENS-653 expression was also a significant predictor of disease-free survival. Together, these findings indicate that ENS-653 could play an oncogenic role in PTC and may serve as a diagnostic and adverse prognostic biomarker for PTC diagnosis.

Recent studies have suggested that a number of lncRNAs could play a critical role in the proliferation of PTC cells [14, 20, 23–30]. Using loss-of-function approaches, we found that ENS-653 knockdown inhibited IHH4 cell proliferation, indicating a role for ENS-653 in regulating proliferation, similar to other lncRNAs. One common mechanism underlying modulation of cell proliferation involves regulation of the cell cycle. As previously reported in various cancers, uncontrolled growth of tumors could be attributable to abnormalities in the regulation of G1 or S phase transition and checkpoints [31, 32]. Our data demonstrated that downregulation of ENS-653 expression might inhibit cell proliferation by inducing G1 phase arrest. However, inhibition of ENS-653 expression had no impact on the apoptosis of PTC cells. These results implied that ENS-653 alters the proliferation of PTC cells via regulation of the cell cycle, but not apoptosis. Thus, the G1-S cell cycle transition might be an effective therapeutic target in PTC. In addition, the present study suggested that ENS-653 downregulation caused decreased levels of cyclin D1, which was similar to the previous reports on the lncRNA PVT1 [30] and BANCR [23]. In those studies, silencing PVT1 or BANCR with siRNA in IHH4 cells also led to a remarkable suppression of cell proliferation, and cell cycle arrest at G0/G1 phase due to inhibition of cyclin D1 [23, 30]. Moreover, both of lncRNA PVT1 and BANCR could be enriched by polycomb enhancer of zeste homolog 2 (EZH2), which is a well-known histone modifier [23, 30]. To date, accumulating evidence has revealed that lncRNAs could regulate multiple biological functions through several processes, including epigenetic modification, transcriptional or post-transcriptional modulation [32]. Histone modification, as well as serving as a scaffold for protein, was involved in the regulation of PTC oncogenesis by lncRNA PVT1 and BANCR [23, 30]. Combined with the results in our study, it remains to be defined in future study that whether histone modification in the promoter region of cyclin D1 was also affected by the suppression of ENS-653.

We further explored the possible signaling pathway by which ENS-653 regulates cell proliferation in PTC. From the available TCGA PTC cohort database, we found that higher ENS-653 expression accompanied with more frequent BRAF (V600E) mutation and BRAF^V600E^ was independently associated with ENS-653 level. Similar to the previous findings [33], the results in this study indicate that ENS-653 is also one of BRAF^V600E^-correlated lncRNAs, which can provide possible candidates for secondary mechanisms of BRAF-induced malignancy in PTC. Notably, mutation in the gene encoding BRAF accounts for the highest frequency of activating mutations in components of the mitogen-activated protein kinase (MAPK) pathway in PTC [34, 35]. Aberrant MAPK signaling and subsequent increases in phosphorylated ERK, JNK and/or p38 kinase drive tumor cell proliferation in a variety of human cancers, particularly in PTC [34, 35]. Here, we demonstrated that downregulation of ENS-653 decreased phosphorylation of ERK1/2 and ERK5 in IHH4 cells. We speculate that this inactivation of ERK1/2 and ERK5 may lead to decreased proliferation of tumor cells. Intriguingly, ERK1/2 and ERK5 but not JNK or p38 might be crucially involved in the proliferation induced by ENS-653 in IHH4 cells. One plausible explanation is that the functions of ERK/MAPK, JNK/MAPK and p38/MAPK might be PTC histotype dependent [17]. However, from the data presented we can only conclude that knock-down of ENS653 negatively regulates MAPK pathway. It is still unclear that whether there is an interaction between ENS-653 and BRAF or other MAPK components as we are unable to perform extra experiments such as genomic sequencing of BRAF hotspot (V600E) from IHH4 cell line or immunoprecipitation (RNA-IP), which is a pitfall of the current study.

The current study has several other limitations. First, all our experiments were performed in vitro, and future animal studies are required to demonstrate the significance of our findings. Second, our study lacks the data regarding overexpression of ENS-653 in vitro and it is also important to investigate the role of MAPK by gain-of-function studies. Third, the precise mechanism underlying how ENS-653 stimulates ERK/MAPK signaling to induce PTC cell proliferation is still unknown and requires additional investigation.

## Conclusion

Here we report a novel lncRNA (ENS-653) whose expression is elevated in human PTC tissues and is associated with higher clinical stages and poor disease-free survival. ENS-653 may promote tumorigenesis via cell cycle regulation and activation of ERK/MAPK signaling in PTC cells. ENS-653 may thus be a viable target for therapeutic intervention, and its elevation may be a valuable indicator of PTC diagnosis and/or prognosis.

## Additional files

Additional file 1: **Table S1.** Patient demographic and baseline characteristics of the study population. **Table S2.** Demographic and baseline characteristics of papillary thyroid cancer patients selected for microarray profiling. **Table S3.** Sequences of lncRNAs. **Table S4.** Correlation of the expression of ENS-653 with clinicopathological features in TCGA PTC cohort. **Table S5.** Regression analysis of association of ENS653 level with BRAF (V600E) mutation or age in TCGA PTC cohort. (ZIP 67 kb)

Additional file 2: : **Figure S1.** Flowchart of microarray analysis or validation study. **Figure S2.** ENS-653 chromosomal location. (ZIP 106 kb)

## Abbreviations

BANCR: BRAF-Activated Long Noncoding RNA
ENS-653: ENST00000539653.1
HT: Hashimoto’s thyroiditis
LncRNA: long non-coding RNA
MAPK: mitogen-activated protein kinase
PTC: papillary thyroid cancer
PTCSC3: Papillary thyroid carcinoma susceptibility candidate 3
RNA-IP: RNA immunoprecipitation
SD: standard deviation
Si-653: siRNA silencing ENS-653
siRNA: small interfering RNA
TCGA: The Cancer Genome Atlas
