# Supplementary material for: LncRNA ENST00000539653 acts as an oncogenic factor via MAPK signalling in papillary thyroid cancer
Source: BMC Cancer. 2019 Apr 2;19:297. doi: 10.1186/s12885-019-5533-4 (PMC6446410; doi:10.1186/s12885-019-5533-4)
Supplement: Supplementary file 1 — Table S1. Patient demographic and baseline characteristics of the study population. Table S2. Demographic and baseline characteristics of papillary thyroid cancer patients selected for microarray profiling. Table S3. Sequences of lncRNAs. Table S4. Correlation of the expression of ENS-653 with clinicopathological features in TCGA PTC cohort. Table S5. Regression analysis of association of ENS653 level with BRAF (V600E) mutation or age in TCGA PTC cohort. (ZIP 67 kb) [file 12885_2019_5533_MOESM1_ESM.zip › Table S5R3.docx]

Table S5: Regression analysis of association of ENS-653 level with BRAF(V600E) mutation or age in TCGA PTC cohort.

|  | BRAF(V600E) mutation | *P* | Age (per 10 years) | *P* |
| --- | --- | --- | --- | --- |
| Unadjusted β, 95%CI | 0.7 (0.6, 0.9) | < 0.001 | -0.1 (-0.1, 0.0) | 0.046 |
| ^†^Adjusted β, 95%CI | 0.7 (0.6, 0.9) | < 0.001 | -0.1 (-0.1, 0.0) | 0.058 |
| ^*^Adjusted β, 95%CI | 0.7 (0.6, 0.9)^a^ | < 0.001 | -0.0 (-0.1, 0.0)^b^ | 0.060 |

^†^ The adjusted β controls for gender.

^a^ The adjusted β controls for gender, age.

^b^ The adjusted β controls for gender, BRAF(V600E) mutation.
