# Supplementary material for: LncRNA ENST00000539653 acts as an oncogenic factor via MAPK signalling in papillary thyroid cancer
Source: BMC Cancer. 2019 Apr 2;19:297. doi: 10.1186/s12885-019-5533-4 (PMC6446410; doi:10.1186/s12885-019-5533-4)
Supplement: Supplementary file 2 — : Figure S1. Flowchart of microarray analysis or validation study. Figure S2. ENS-653 chromosomal location. (ZIP 106 kb) [file 12885_2019_5533_MOESM2_ESM.zip › Figure S1R3.docx]

Figure S1. Flowchart of patients selection

Patients used for qRT-PCR validation (n=86)

Patients used for microarray study (n=4)

Patients involved in analysis (n=90)

Excluded (n=10)

Patients with a positive history of other malignant tumors (n=5);

Patients diagnosed with other severe diseases (n=3);

Patients with a history of ^131^I therapy (n=2)

Patients with pathologically confirmed papillary thyroid cancer (n=100)
