# Supplementary figures and images for: LncRNA ENST00000539653 acts as an oncogenic factor via MAPK signalling in papillary thyroid cancer
Source: BMC Cancer. 2019 Apr 2;19:297. doi: 10.1186/s12885-019-5533-4 (PMC6446410; doi:10.1186/s12885-019-5533-4)

**
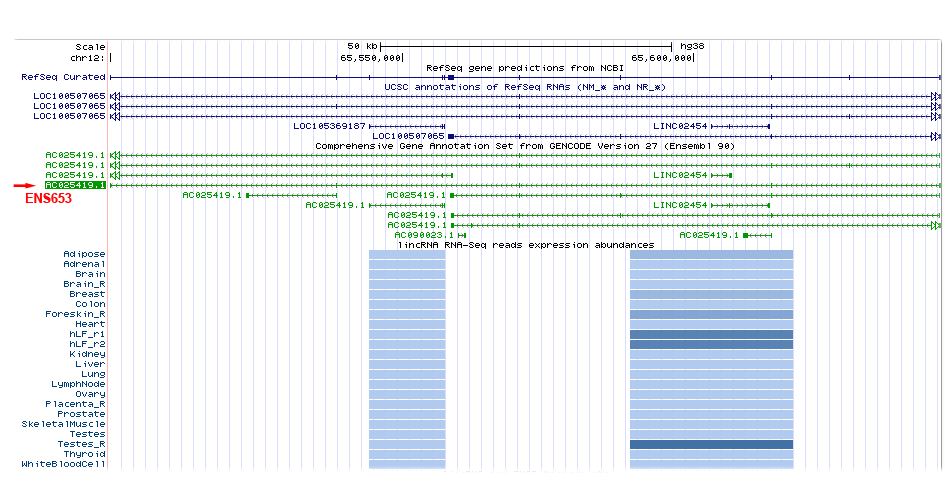
**

**Figure S2.** ENS-653 chromosomal location.

Supplement: Supplementary file 2 — : Figure S1. Flowchart of microarray analysis or validation study. Figure S2. ENS-653 chromosomal location. (ZIP 106 kb) [file 12885_2019_5533_MOESM2_ESM.zip › Figure S2R3.docx]
